# Diagnosis and Management of Immune Thrombocytopenia in Paediatrics: A Comprehensive Review

**DOI:** 10.7759/cureus.69635

**Published:** 2024-09-18

**Authors:** Yash Thakur, Revat J Meshram, Amar Taksande

**Affiliations:** 1 Pediatrics, Jawaharlal Nehru Medical College, Datta Meghe Institute of Higher Education and Research, Wardha, IND

**Keywords:** autoimmune disorders, childhood itp treatment, immune thrombocytopenia (itp), pediatric hematology, platelet count management, thrombocytopenia diagnosis

## Abstract

Immune thrombocytopenia (ITP) in paediatric patients is a complex and heterogeneous disorder characterized by isolated thrombocytopenia and an increased risk of bleeding. The diagnosis of ITP involves a careful exclusion of other causes of thrombocytopenia, supported by clinical evaluation and laboratory findings. Management strategies have evolved significantly, emphasizing individualized treatment approaches based on disease severity, bleeding risk, and patient-specific factors. This comprehensive review provides an in-depth analysis of the current diagnostic criteria, including the role of novel biomarkers and genetic testing in distinguishing ITP from other haematological disorders. We also explore the latest therapeutic options, ranging from observation and first-line treatments such as corticosteroids and intravenous immunoglobulin (IVIG) to second-line therapies, including thrombopoietin receptor agonists and immunosuppressive agents. The review addresses the challenges of managing chronic ITP in pediatric patients, focusing on balancing treatment efficacy with the potential side effects and long-term outcomes. Additionally, we discuss the emerging role of personalized medicine in optimizing care for children with ITP, highlighting recent advances in targeted therapies and the potential for future research to refine diagnostic and treatment paradigms to refine diagnostic and treatment paradigms further.

## Introduction and background

Immune thrombocytopenia (ITP) is a haematological disorder characterized by an isolated decrease in platelet count, leading to an increased risk of bleeding. It is one of the most common causes of thrombocytopenia in children, with an incidence of approximately one in 20,000 children per year [1]. ITP can present as either acute or chronic, with the majority of paediatric cases being acute and self-limiting. However, a significant proportion of children develop chronic ITP, persisting beyond 12 months from diagnosis [2]. The pathophysiology of ITP involves the immune system's production of autoantibodies that target platelet antigens, resulting in platelet destruction predominantly in the spleen [3]. Despite its relatively benign course in most cases, the unpredictable nature of ITP can lead to severe hemorrhagic complications, necessitating vigilant diagnosis and management.

Early diagnosis and effective management of ITP are critical to minimizing the risk of severe bleeding and improving patient outcomes. In paediatric patients, the challenge lies in distinguishing ITP from other causes of thrombocytopenia, such as bone marrow disorders, which may require different therapeutic approaches [4]. A prompt and accurate diagnosis allows for the appropriate stratification of patients based on bleeding risk and the initiation of treatment when necessary. Moreover, effective management, which may range from observation to pharmacologic intervention, plays a vital role in preventing chronicity and reducing the impact of ITP on the child's quality of life [5]. The evolution of treatment strategies, including corticosteroids, intravenous immunoglobulin (IVIG), and thrombopoietin receptor agonists, has improved the prognosis for children with ITP. Still, challenges remain in optimizing therapy and balancing efficacy with potential side effects [6].

This comprehensive review aims to provide an in-depth examination of the current landscape in the diagnosis and management of ITP in paediatric patients. We aim to consolidate recent advances in understanding ITP pathophysiology, highlight the latest diagnostic approaches, and evaluate the efficacy and safety of emerging treatment modalities. This review seeks to inform clinical practice by synthesizing current evidence, offering guidance on best practices for managing paediatric ITP and identifying areas where further research is needed. This review is intended for healthcare professionals involved in the care of children with ITP and researchers interested in the future directions of ITP management.

## Review

Search methodology

For the comprehensive review of the diagnosis and management of immune thrombocytopenia (ITP) in paediatrics, we employed a rigorous search methodology to ensure a thorough evaluation of the current literature. We conducted a systematic search across multiple electronic databases, including PubMed, Embase, Cochrane Library, and Web of Science, using keywords and medical subject headings related to "immune thrombocytopenia," "pediatrics," "diagnosis," and "management." We included studies published from January 2000 to the present to capture the most recent advancements and treatment approaches. The search was supplemented by reviewing references from relevant articles and guidelines to identify additional studies. Inclusion criteria encompassed peer-reviewed articles, clinical trials, case reports, and reviews focusing on paediatric ITP. Articles were screened for relevance based on abstract and full-text reviews, and data were extracted and synthesized to provide a comprehensive overview of current diagnostic practices, treatment modalities, and management strategies in paediatric ITP.

Pathophysiology of paediatric immune thrombocytopenia

Mechanisms of Platelet Destruction

The primary mechanism of thrombocytopenia in ITP involves the immune-mediated destruction of platelets. Autoantibodies, particularly those targeting glycoproteins on the platelet surface, such as GPIIb/IIIa and GPIb/IX, play a pivotal role in this process. These autoantibodies are produced by autoreactive B cells and subsequently bind to platelets, marking them for destruction by macrophages in the spleen and liver through Fc receptor-mediated phagocytosis [7]. In addition to phagocytosis, platelet destruction may also occur through complement activation, where the classical complement pathway is triggered, leading to opsonization and further clearance of platelets [8].

Impaired Platelet Production

Beyond the enhanced peripheral destruction, impaired platelet production is also a critical factor in the pathogenesis of ITP. Megakaryocytes, the precursor cells responsible for platelet production in the bone marrow, are adversely affected by ITP. Studies have shown that autoantibodies and cytotoxic T cells may target megakaryocytes, leading to their apoptosis and reduced platelet production [9]. Moreover, inflammatory cytokines such as interleukin-1β (IL-1β) and tumour necrosis factor-alpha (TNF-α) have been implicated in the suppression of megakaryocyte maturation and platelet release [10]. The combination of increased platelet destruction and inadequate compensatory production results in the sustained thrombocytopenia observed in ITP.

Genetic and Environmental Influences

The interplay between genetic predisposition and environmental triggers is also crucial in the development of ITP. Genetic factors, such as polymorphisms in immune regulatory genes (e.g., CTLA-4, PTPN22), have been associated with an increased risk of developing ITP, suggesting a hereditary component to the dysregulation of immune tolerance [11]. Environmental factors, including viral infections and vaccinations, have also been identified as potential triggers for ITP. Molecular mimicry, where viral antigens resemble platelet surface proteins, may lead to the production of cross-reactive antibodies, initiating the autoimmune response [12]. Furthermore, epigenetic modifications, such as DNA methylation changes in immune-related genes, may also contribute to the onset and progression of the disease [13].

Clinical presentation and diagnosis

Signs and Symptoms of ITP in Children

In children, immune thrombocytopenia (ITP) typically presents with a sudden onset of petechiae, purpura, and mucosal bleeding, including epistaxis and gingival bleeding. Severe haemorrhage, such as gastrointestinal or intracranial bleeding, is rare but can occur, especially in cases of profound thrombocytopenia (platelet count < 10,000/µL) [14]. Children are often otherwise healthy, with regular physical examinations aside from bleeding signs. Unlike adults, splenomegaly, lymphadenopathy, and systemic symptoms are generally absent in paediatric ITP, which helps differentiate it from other hematologic disorders [15].

Differential Diagnosis

The differential diagnosis for ITP in children is broad and includes conditions that also present with thrombocytopenia or similar bleeding manifestations. Key conditions to consider include the following:

*Viral infections*: Viral infections significantly contribute to the clinical presentation of immune thrombocytopenia (ITP) in paediatric patients. Common viral triggers, such as Epstein-Barr virus (EBV), cytomegalovirus (CMV), and varicella-zoster virus (VZV), can initiate an immune response that mistakenly targets platelets, leading to their destruction. Clinically, children with ITP often present with sudden onset of petechiae, purpura, and mucosal bleeding, which may be exacerbated following a viral illness. Diagnosing ITP in the context of a recent viral infection requires careful evaluation of the patient's history, physical examination, and laboratory findings, including a complete blood count (CBC) revealing isolated thrombocytopenia and exclusion of other potential causes. Serological tests may identify specific viral infections, aiding the differentiation between primary ITP and secondary thrombocytopenia related to the underlying viral condition [16].

*Leukaemia*: In pediatric patients, leukaemia must be considered in the differential diagnosis of immune thrombocytopenia (ITP) due to its potential to present with similar clinical features, such as isolated thrombocytopenia, petechiae, and bruising. Unlike ITP, leukaemia often presents with additional signs like hepatosplenomegaly, lymphadenopathy, and abnormal white blood cell counts, which may include blasts on peripheral blood smears. Bone marrow examination is crucial in distinguishing leukaemia from ITP, as it reveals leukemic blasts rather than the increased megakaryocytes typical of ITP. Early recognition and accurate differentiation between these conditions are essential to avoid delays in diagnosing and treating leukaemia, which requires a vastly different therapeutic approach [17].

*Bone marrow disorders*: Bone marrow disorders in the context of pediatric immune thrombocytopenia (ITP) are critical to consider during diagnosis, as they can mimic or coexist with ITP. Clinically, these disorders may present with persistent thrombocytopenia, pancytopenia, or atypical features such as significant lymphadenopathy or hepatosplenomegaly, which are uncommon in isolated ITP. Bone marrow examination is typically reserved for cases where initial treatments fail or concerning clinical features reveal potential underlying conditions like leukaemia, myelodysplastic syndromes, or aplastic anaemia. Accurate differentiation is crucial, as these disorders require distinct management strategies compared to ITP [18].

*Systemic lupus erythematosus* (*SLE*): Systemic lupus erythematosus (SLE) is a significant autoimmune condition that can manifest with immune thrombocytopenia (ITP) in paediatric patients. SLE-associated ITP often presents with a range of clinical features, including mucocutaneous bleeding, petechiae, and bruising, which can be indistinguishable from primary ITP. However, additional systemic symptoms such as malar rash, arthritis, renal involvement, and positive antinuclear antibodies (ANA) should prompt consideration of SLE in the differential diagnosis. Diagnosing SLE-associated ITP requires a thorough clinical evaluation and serological testing, including ANA, anti-double-stranded DNA (anti-dsDNA) antibodies, and complement levels, to differentiate it from primary ITP. Early recognition and appropriate management are crucial as SLE-associated ITP may require a different therapeutic approach, including immunosuppressive agents, to control the underlying autoimmune process and prevent disease flares [19].

Diagnostic Workup

The diagnostic workup for ITP in children includes a detailed history and physical examination, focusing on the onset and severity of bleeding symptoms, recent infections, medication use, and family history of bleeding disorders. Laboratory tests should include a complete blood count (CBC) with a peripheral smear, typically showing isolated thrombocytopenia with large platelets and no other haematologic abnormalities [20]. Further tests may be indicated based on the clinical context:

*Bone marrow aspiration/biopsy*: Bone marrow aspiration and biopsy are valuable tools in the clinical presentation and diagnosis of paediatric immune thrombocytopenia (ITP), particularly in cases where the diagnosis is uncertain or when atypical features are present, such as unexplained anaemia, leukopenia, or systemic symptoms. While typically not required for the diagnosis of primary ITP, these procedures help exclude other serious haematologic conditions like leukaemia or myelodysplastic syndromes, which can present with similar clinical features. In ITP, bone marrow findings generally reveal normal or increased numbers of megakaryocytes, reflecting an increased platelet production response. The decision to perform a bone marrow examination should be individualized, guided by clinical judgement, and is usually reserved for complex or refractory cases [21].

*Viral serologies*: Viral serologies play a critical role in the clinical presentation and diagnosis of immune thrombocytopenia (ITP) in paediatric patients, as several viral infections, including Epstein-Barr virus (EBV), cytomegalovirus (CMV), and hepatitis C, have been implicated in the pathogenesis of ITP. These infections can trigger an autoimmune response, leading to the destruction of platelets. Testing for viral serologies is essential in the diagnostic workup to identify the underlying infections that may contribute to or exacerbate thrombocytopenia. Early identification of viral etiologies not only aids in distinguishing primary ITP from secondary causes but also influences the management strategy, as addressing the underlying viral infection can lead to the resolution of thrombocytopenia or alter the therapeutic approach [22].

*Autoantibody tests*: Autoantibody tests play a crucial role in the clinical presentation and diagnosis of immune thrombocytopenia (ITP) in paediatric patients. These tests, including assays for antiplatelet antibodies, can help distinguish primary ITP from other autoimmune disorders where thrombocytopenia is secondary. Although the presence of autoantibodies against platelet glycoproteins, such as GPIIb/IIIa or GPIb/IX, is not always definitive, it supports an immune-mediated mechanism of platelet destruction. However, it is important to note that these tests are not routinely performed due to variability in sensitivity and specificity, and their results should be interpreted in conjunction with clinical findings and other diagnostic tests [23].

Diagnostic Criteria and Classification

The diagnosis of ITP is primarily one of exclusion, confirmed by the presence of isolated thrombocytopenia (platelet count < 100,000/µL) with normal bone marrow function and the absence of other causes of thrombocytopenia [24]. Table 1 shows that ITP is classified based on the duration of the disease.

**Table 1:** Immune thrombocytopenia (ITP) classification

ITP Classification	Duration
Newly diagnosed ITP	Diagnosed within three months of onset
Persistent ITP	Lasting between 3 and 12 months
Chronic ITP	Persistent thrombocytopenia beyond 12 months

This classification helps guide management and predict the likelihood of spontaneous remission. Chronic ITP is less common in children, with most cases resolving within the first year without significant long-term consequences [25].

Management strategies

Initial Management of Newly Diagnosed ITP

*Observation vs. intervention*: In paediatric patients newly diagnosed with immune thrombocytopenia (ITP), the decision between observation and active intervention depends on the severity of thrombocytopenia, bleeding symptoms, and overall clinical condition. The observation is often preferred for asymptomatic or mildly symptomatic children, especially when platelet counts are above 20,000-30,000/μL [15]. Studies have demonstrated that many children with ITP experience spontaneous recovery without the need for pharmacologic intervention [26].

*First-line therapies*: When intervention is required, first-line therapies include corticosteroids, intravenous immunoglobulin (IVIG), and anti-D immunoglobulin. Corticosteroids, such as prednisone, are commonly used due to their efficacy in rapidly increasing platelet counts; however, their use is limited by side effects, particularly with prolonged administration [27]. IVIG is another first-line option, often used when rapid platelet count elevation is necessary, such as in cases of significant bleeding or before surgery. The mechanism involves immunomodulation, though its effect is usually transient [28]. Anti-D immunoglobulin, an option for Rh-positive, non-splenectomized patients, induces mild hemolysis, thereby diverting autoantibodies away from platelets [12].

Management of Chronic ITP

*Second-line treatments*: For paediatric patients whose ITP becomes chronic (lasting more than 12 months), second-line treatments are considered when first-line therapies fail to maintain safe platelet levels or when side effects become intolerable. Rituximab, a monoclonal antibody against CD20, depletes B cells and reduces autoantibody production. Clinical studies have shown their effectiveness in achieving durable remission in a subset of paediatric patients [29]. Thrombopoietin receptor agonists (TPO-RAs), such as eltrombopag and romiplostim, stimulate platelet production and have shown promising results in children with chronic ITP, providing an alternative to immunosuppressive therapies [30].

*Role of splenectomy and other surgical options*: Splenectomy, the surgical removal of the spleen, has been a traditional treatment for chronic ITP, offering potential long-term remission. However, due to its invasive nature and associated risks, such as infection and thrombosis, it is generally reserved for cases where other treatments have failed [31]. Laparoscopic splenectomy is preferred over open surgery due to its minimally invasive approach and reduced recovery time [32]. Other surgical options, such as partial splenectomy or splenic artery embolization, are less common and typically considered in special circumstances [33].

Management of Refractory ITP

*Options for treatment-resistant cases*: Refractory ITP, defined as a lack of response to standard therapies, presents a significant challenge in management. Treatments like high-dose corticosteroids, rituximab, or combination therapies with immunosuppressive agents like mycophenolate mofetil or cyclosporine may be considered [19]. These approaches aim to modulate the immune system further and control platelet destruction.

*Experimental therapies and clinical trials*: Ongoing research into the pathophysiology of ITP has led to the development of experimental treatments targeting various immune pathways. For instance, fostamatinib, a spleen tyrosine kinase (Syk) inhibitor, has shown promise in adult clinical trials and may be extended to paediatric use [34]. Clinical trials remain a critical avenue for accessing novel therapies, and participation in these trials can be considered for children with refractory ITP [35].

Special considerations in paediatric ITP

ITP in Infants and Young Children

Immune thrombocytopenia (ITP) in infants and young children presents distinct clinical challenges compared to older paediatric populations. Typically, ITP in this age group is acute and self-limiting, with a spontaneous remission rate of approximately 70-80% within the first six months of diagnosis [14]. Management often focuses on observation rather than immediate intervention unless the child exhibits significant bleeding symptoms. In cases where treatment is necessary, intravenous immunoglobulin (IVIG) and corticosteroids are commonly used, with anti-D immunoglobulin reserved for specific cases [15]. The differential diagnosis in this age group is crucial, as other causes of thrombocytopenia, such as congenital platelet disorders or infections, must be excluded [36].

Adolescents with ITP

Adolescents with ITP present unique challenges due to the interplay of disease and developmental factors. Unlike younger children, ITP in adolescents is more likely to become chronic, with approximately 20-30% of cases persisting beyond 12 months [37]. The psychological impact of chronic ITP, including concerns about physical appearance due to bruising and limitations on physical activities, can significantly affect the quality of life in this age group. Management strategies must, therefore, balance the need for effective treatment to minimize the impact on daily life and psychological well-being. Therapies such as thrombopoietin receptor agonists (TPO-RAs) are increasingly used in this population because they sustain platelet counts with fewer side effects than corticosteroids [38].

ITP and Vaccination

The relationship between ITP and vaccination, particularly with the MMR (measles, mumps, and rubella) vaccine, is well-recognized. Studies have shown a small but significant risk of developing ITP following MMR vaccination, particularly in the first six weeks post-vaccination, with an estimated incidence of one case per 40,000 doses administered [26]. However, the benefits of vaccination far outweigh the risks, and children with a history of ITP are not typically contraindicated from receiving standard vaccines [39]. It is recommended that healthcare providers monitor platelet counts in children with a history of ITP following vaccination, especially in those who have had severe or chronic ITP [40].

Management of ITP during Pregnancy

Managing ITP during pregnancy requires careful consideration due to the potential risks to both the mother and the fetus. While ITP in pregnancy is often mild and manageable, the condition can exacerbate, especially during the third trimester and delivery, necessitating intervention to ensure safe platelet levels during labour. IVIG and corticosteroids remain the mainstays of treatment, as they are generally safe for both the mother and fetus [9]. The risk of neonatal thrombocytopenia due to transplacental passage of antiplatelet antibodies is another concern and close monitoring of the newborn's platelet count is essential [12]. The decision to treat ITP during pregnancy should be individualized, taking into account the severity of thrombocytopenia, the presence of bleeding symptoms, and the risks associated with therapy.

Monitoring and long-term outcomes

Monitoring Disease Progression

Monitoring the progression of immune thrombocytopenia (ITP) in paediatric patients involves regular assessments of platelet counts, bleeding symptoms, and overall clinical status. Key strategies include periodic blood tests to monitor platelet levels and clinical scoring systems to assess disease severity and response to treatment [9]. In cases of chronic ITP, routine evaluations every three to six months are recommended to adjust treatment plans and manage complications [23]. Advanced monitoring techniques, such as imaging studies, may be necessary for evaluating the spleen size in patients with suspected splenomegaly, which can influence treatment decisions [31].

Complications of ITP and Its Treatment

Complications associated with ITP and its treatment can be significant and multifaceted. Bleeding complications remain a primary concern, especially in cases of severe thrombocytopenia. Long-term corticosteroid use, a standard treatment for ITP, can lead to adverse effects such as growth retardation, osteoporosis, and increased susceptibility to infections [41]. Additionally, therapies like intravenous immunoglobulin (IVIG) and anti-D immunoglobulin can cause allergic reactions and hemolytic anaemia [19]. The risk of developing secondary ITP due to underlying conditions, including autoimmune disorders or malignancies, must also be considered [23].

Quality of Life and Psychosocial Impact

The impact of ITP on a child's quality of life can be profound, affecting physical, emotional, and social well-being. Children with chronic ITP may experience limitations in physical activity, increased school absenteeism, and social stigma related to their condition. Psychosocial stressors, including anxiety and depression, are also every day among these patients and can be exacerbated by the chronic nature of the disease and its treatments [41]. To address these issues effectively, it is essential to incorporate multidisciplinary support, including psychological counselling and educational interventions [42].

Prognosis and Predictors of Outcomes

The prognosis of paediatric ITP varies widely based on factors such as age at diagnosis, severity of thrombocytopenia, and response to initial treatments. Children with acute ITP often have a favourable prognosis, with many achieving remissions within six to 12 months [23]. Conversely, chronic ITP may lead to prolonged management and an increased risk of complications. Predictors of poorer outcomes include persistent severe thrombocytopenia, failure to respond to first-line treatments, and secondary conditions [9]. Regular follow-up and individualized treatment plans are crucial to improving long-term outcomes and minimizing the risk of complications [19].

Recent advances and future directions

Recent advances in diagnosing and managing immune thrombocytopenia (ITP) in paediatric patients have significantly improved our understanding and treatment of this complex disorder. The development of more sensitive diagnostic assays, including platelet-specific autoantibody tests and advanced imaging techniques, has enhanced our ability to diagnose and monitor ITP accurately. New insights into the pathophysiology of ITP, driven by research on immune dysregulation and genetic predispositions, have led to the introduction of targeted therapies, such as thrombopoietin receptor agonists and novel immunomodulatory agents, which offer more effective and personalized treatment options. Future directions involve further refinement of these therapies, with ongoing clinical trials exploring combination treatments and novel agents to address acute and chronic ITP more effectively. Additionally, integrating genetic and immunological biomarkers into clinical practice promises to improve risk stratification and tailor management strategies, paving the way for individualized treatment approaches and potentially enhancing outcomes for paediatric patients with ITP.

## Conclusions

ITP in paediatric patients presents a unique challenge due to its variable aetiology, clinical presentation, and response to treatment. This comprehensive review highlights the complexity of diagnosing and managing ITP in children, emphasizing the importance of a thorough evaluation to distinguish between primary and secondary causes. Early diagnosis and tailored management strategies are crucial to improve outcomes and minimize complications. Current management approaches range from observational strategies in asymptomatic cases to medical treatments, such as corticosteroids and intravenous immunoglobulin, and, in more severe instances, splenectomy or other surgical interventions. The evolving landscape of treatment options, including novel therapies and emerging biologics, offers new hope for patients with refractory ITP.

The review underscores the need for a multidisciplinary approach involving haematologists, immunologists, and other specialists to provide comprehensive care. Regular follow-up and individualized treatment plans based on disease severity, patient age, and response to therapy are essential for optimizing management and improving the quality of life for paediatric patients with ITP. Future research should focus on better understanding the pathophysiology of ITP, refining diagnostic criteria, and evaluating long-term outcomes of various treatment strategies. As our knowledge of ITP continues to advance, it is crucial to integrate new findings into clinical practice to enhance the care and outcomes for children affected by this condition.
